# A Pilot Predictive Model for Indirect Assessment of Suicidal Ideation

**DOI:** 10.1192/j.eurpsy.2024.1644

**Published:** 2024-08-27

**Authors:** P. Rus Prelog, T. Matić, P. Pregelj, A. Sadikov

**Affiliations:** ^1^Centre for Clinical Psychiatry, University Psychiatric Clinic Ljubljana; ^2^Medical Faculty, Universtity of Ljubljana; ^3^Artificial Intelligence Laboratory, Faculty of Computer and Information Science, University of Ljubljana, Ljubljana, Slovenia

## Abstract

**Introduction:**

In recent years, there has been a concerning increase in suicidal thoughts and, in some countries, completed suicides, amplified by the COVID-19 pandemic. Screening for suicidal ideation (SI) in the general population is limited due to ethical, effectiveness, and feasibility concerns. Identifying individuals at risk of suicide remains a complex challenge. Our study aimed to develop a predictive model using COVID-19 data, gathering psychometric information from 1790 respondents in Slovenia via an online survey conducted between July 2020 and December 2020, with a second wave of data (ne=1200) collected from January 2022 to February 2022.

**Objectives:**

With 9.7% of respondents reporting recent SI in the first wave of data, our primary goal was to estimate SI indirectly using SIDAS. We examined changes in habits, demographics, coping strategies, and satisfaction in key life aspects to discreetly identify potential risk factors.

**Methods:**

We employed four machine learning algorithms (logistic regression, random forest, XGBoost, and support vector machines) and assessed model performance using the area under the receiver operating characteristic curve (AUC). Initial assessment used a held-out dataset, followed by validation with a new cohort of 1,200 users from the late COVID-19 period.

**Results:**

Logistic regression, random forest, and XGBoost achieved comparable AUCs, reaching 0.83 on unseen data. Our analysis revealed significant associations between Brief-COPE subscales and SI. Self-Blame emerged as a strong SI indicator, followed by increased Substance Use, reduced Positive Reframing, Behavioral Disengagement, dissatisfaction with relationships, and younger age, in both 2020 and 2022 models. The model consistently performed well, even with varying population characteristics.

**Conclusions:**

These results suggest that SI presence can be reasonably estimated using selected indicators, offering promise for developing an indirect screening tool without explicit questioning about suicidal thoughts. However, individuals flagged as at-risk should undergo clinical examination, as this model serves as an initial step in identifying SI risk factors in the context of the stressful event’s (COVID-19 pandemic) impact on mental health.

**Disclosure of Interest:**

None Declared

